# Circulating CD56+ NKG2D+ NK cells and postoperative fertility in ovarian endometrioma

**DOI:** 10.1038/s41598-020-75570-z

**Published:** 2020-10-29

**Authors:** Zhi-Qin Liu, Mei-Yin Lu, Bin Liu

**Affiliations:** 1 Department of Obstetrics and Gynecology, Shenzhen Baoan Women’s and Children’s Hospital, Jinan University, Shenzhen, 518102 Guangdong China; 2 Department of Biobank, Shenzhen Baoan Women’s and Children’s Hospital, Jinan University, Shenzhen, 518102 Guangdong China

**Keywords:** Biomarkers, Outcomes research, Lymphocytes

## Abstract

The current biomarkers for postoperative fertility assessment caused by ovarian endometrioma (OE) are insufficient. The present study hypothesized that the peripheral lymphocyte subpopulation can be used as a candidate biomarker of postoperative infertility in OE. The association of the number of circulating CD4/CD8 T, NK, and γδ T cells with postoperative fertility was assessed in 33 OE patients aged 20 ~ 40 years between June 2018 and January 2019. Concomitantly, 68 healthy female subjects were recruited. The changes in the baseline immune characteristics between the two groups were compared. The data demonstrated significant differences in the ratio of CD4/CD8 T cells and the number of CD56+ NKG2D+ NK cells and γδ T cells between OE patients and control subjects. The patients were followed-up till December 2019 and the number of CD56+ NKG2D+ NK cells in the cases was a significant predictor for postoperative fertility as determined by different COX regression models (crude HR = 0.220, 95% CI = 0.059–0.822; adjusted HR = 0.127, 95% CI = 0.024–0.675). A significant delay to successful pregnancy was noted in OE patients (median time, 173 vs. 99 days, log-rank *P* = 0.013). The present findings suggested that CD56+ NKG2D+ NK cells are a candidate biomarker of postoperative fertility in OE patients. Larger population studies are warranted.

## Introduction

Endometriosis is an important cause of infertility and is highly prevalent in reproductive women^1^. In addition to infertility, endometriosis is also associated with pelvic pain, which is caused by repeated bleeding occurring in the ectopic endometrium in every menstrual cycle due to the growth of the endometrioid tissue outside the pelvis^1^. The most common and important type of endometriosis is ovarian endometrioma (OE), accounting for 17–44% of endometriosis^2^. OE is characterized by an ovarian cyst filled with chocolate-like fluid, which is produced by repeated bleeding in the ovary^3^. It causes chronic pelvic pain, difficulty in sexual intercourse^2^ and infertility^4^. This is partly due to a significantly lower reproductive capacity of OE patients compared with that of the healthy subjects^5^.

Laparoscopy coupled with histological confirmation is the gold standard for the diagnosis of OE^6^. However, a mean latency of 7–11 years from the onset of symptoms to the definitive diagnosis is required by laparoscopy, which may have significant consequences in terms of disease progression, including the severity of pelvic pain that may lead to hysterectomy and oophorectomy^6^. Increasing evidence has suggested that the malignant transformation of OE cells can lead to ovarian cancer^6,7^. In addition, a surgical diagnosis has multiple drawbacks, including organ damage, hemorrhage, infection, adhesion formation and anesthetic complications^6^. Therefore, the application of new non-invasive biomarkers may be helpful for earlier diagnosis and prevention of the unwanted sequelae. Moreover, it can reduce organ damage caused by surgery.

Recent studies have reported that surgical treatment can significantly improve the prognosis of fertility complications caused by endometriosis^5,8^. However, certain side effects of surgery, including vaginal bleeding and gastrointestinal symptoms have been documented in specific patients^5,8,9^. In addition, infertility induced by ovarian surgery and the occurrence of recurrent OE should also be considered prior to operation^9^. Moreover, the current biomarkers used for postoperative fertility in OE subjects are insufficient. Therefore, the use of non-invasive biomarkers that can assess disease status prior to the operation is warranted in OE patients in order to optimize the selection of treatment.

Despite the prevalence of OE, its etiology and the exact causal relationship between OE and infertility is unclear^10^. Several proposed pathogenic theories have been documented, such as retrograde menstruation, coelomic metaplasia and Müllerian remnants^11^. Among them, the retrograde menstruation hypothesis is considered as the most convincing model. It can be summarized as follows: endometrial fragments reach and implant onto the ovary via transtubal retrograde flow. Subsequently, they proliferate and cause chronic inflammation and adhesion, which leads to the occurrence of OE^11^. Therefore, OE is often considered an inflammatory disease^12^. Notably, dysfunction of various lymphocyte subpopulations is pivotal for the pathogenesis of endometriosis^13,14^. For example, the imbalance of helper T cells (Th), B cells and Natural Killer (NK) cells, can lead to inflammation and further development of the ovarian lesions in OE^12^. Since blood biomarkers are considered optimal non-invasive diagnostic indicators of endometriosis^7^, it is feasible to use the peripheral lymphocyte subset in the diagnosis and prognosis of OE.

Furthermore, the increase of inflammatory cytokines, growth factors and angiogenic factors secreted by various lymphocyte subpopulations in OE patients can alter the pelvic environment and seriously affect their fertility^15^. The dysfunction of these lymphocytic subpopulations affects the development of follicles and ovulation, impeding sperm capacity, oocyte-sperm interaction and embryo implantation, which in turn increases the risk of infertility and other female reproductive events^16,17^. For example, the number of uterine NK progenitor cells, which play a critical role early in gestation, is markedly decreased in endometriosis patients who proceed to failed embryo implantation and may consequently be considered an available predictor of implantation success^18^. In addition, both Th cells^19,20^ and γδ T cells^21^ have been shown to be involved in the etiology of infertility. Taken together, the aforementioned studies suggest that lymphocyte subpopulations are candidate biomarkers for infertility. OE is caused by an inflammatory reaction noted in multiple organs including ovary and uterus^12^, which are both associated with infertility. Therefore, the peripheral lymphocyte subpopulations may be valuable to predict the infertility of OE cases. In addition, the use of the peripheral lymphocyte subset as a noninvasive biomarker is more convenient for clinical applications. However, a limited number of studies have reported the association between peripheral immune cells and postoperative infertility in OE. We hypothesized that peripheral lymphocyte subpopulations are candidate biomarkers of postoperative infertility in OE.

In the present study, we investigated the association of circulating CD4/CD8 T, NK, and γδ T cells with postoperative fertility in OE. Briefly, we analyzed initially the peripheral lymphocyte subsets in 33 OE cases prior to operation, which were compared with 68 healthy control subjects. Subsequently, the fertility status following operation in these OE cases was followed up and the association of statistical significant immune indicators with the rate and median time of postoperative infertility in OE was analyzed.

## Results

### Clinical and immune characteristics of the OE patients at baseline

The characteristics of the subjects are summarized in Table 1 and Suppl. Table 1. Overall, the differences in the distributions of age, body mass index, early menarche, menstrual cycle and parity between the cases and controls were not statistically significant (all *P* values < 0.05). However, the OE patients were prone to develop anemia, lymphopenia and a longer menstrual duration compared with that of the control subjects (all *P* values < 0.05).

**Table 1:** Clinical and demographic characteristics of OE patients (median, IQR).

Characteristic	OE patients (n = 33)	Controls (n = 68)	P*
Age (years)	30 (27, 33)	30 (27, 32)	0.681
Body mass index (Kg/m^2^**)**	20.2 (19.0, 21.0)	20.4 (19.0, 22.4)	0.328
Age at menarche ≤ 14 years (n,%)	24 (72.7)	47 (69.1)	0.780
Menstrual cycle (days)	30 (30, 30)	30 (29, 31)	0.151
Menstrual duration (days)	7 (5, 7)	6 (6, 8)	**0.043**
Nulliparous (n,%)	14 (42.4)	35 (51.5)	0.394
Dysmenorrhea (n,%)	16 (48.5)	–	
Bilateral OE (n,%)	6 (18.2)	–	
OE diameter ≥ 7.0 cm (n, %)	12 (36.4)	–	
Complication (n, %)			
Deep infiltrating endometriosis	6 (18.2)	–	
Pelvic endometriosis	18 (54.6)	–	
Blood tests			
Hemoglobin (g/L)^&^	126 (120, 127)	134 (127, 139)	** < 0.001**
Leukocytes (10^9^/L)^&^	6.12 (4.95, 6.86)	5.90 (5.26, 6.76)	0.778
Lymphocytes (10^9^/L)^&^	1.55 (1.30, 2.01)	2.04 (1.77, 2.30)	** < 0.001**
Erythrocytes (10^12^/L)^&^	4.44 (4.25, 4.57)	4.42 (4.26, 4.72)	0.945
Anti-Mullerian hormone (ng/L)^$^	4.65 (3.15, 5.00)	–	
Cancer antigen 125 (U/ml)^&^	62.7 (41.3, 64.9)		
Surgical approach (n, %)			
Laparoscope	21 (63.6)	–	
Hysteroscope	2 (6.1)	–	
Laparoscope & hysteroscope	10 (30.3)	–	
Follow-up (days), median (IQR, total)	435 (209, 502, 11871)	453 (96, 485, 20632)	0.141
Number of pregnants after operation (n, %)#	12 (36.4)	35 (51.5)	0.153
Time to pregnancy after operation (days)#	127 (32, 240)	135 (68, 258)	0.591

OE: Ovarian endometrioma; IQR = interquartile range; * Mann–Whitney U tests; $, missing two patients; &, missing three patients. Other variables had no missing data. # The pregnant values in the controls were collected during the study period. The rate of pregnancy between cases and controls during the study period were compared by chi-square test, and the time to pregnancy between these two groups were analyzed by log-rank test. The value in bold was *P* value less than 0.05, which had certain statistical significance.

**Figure 1:**
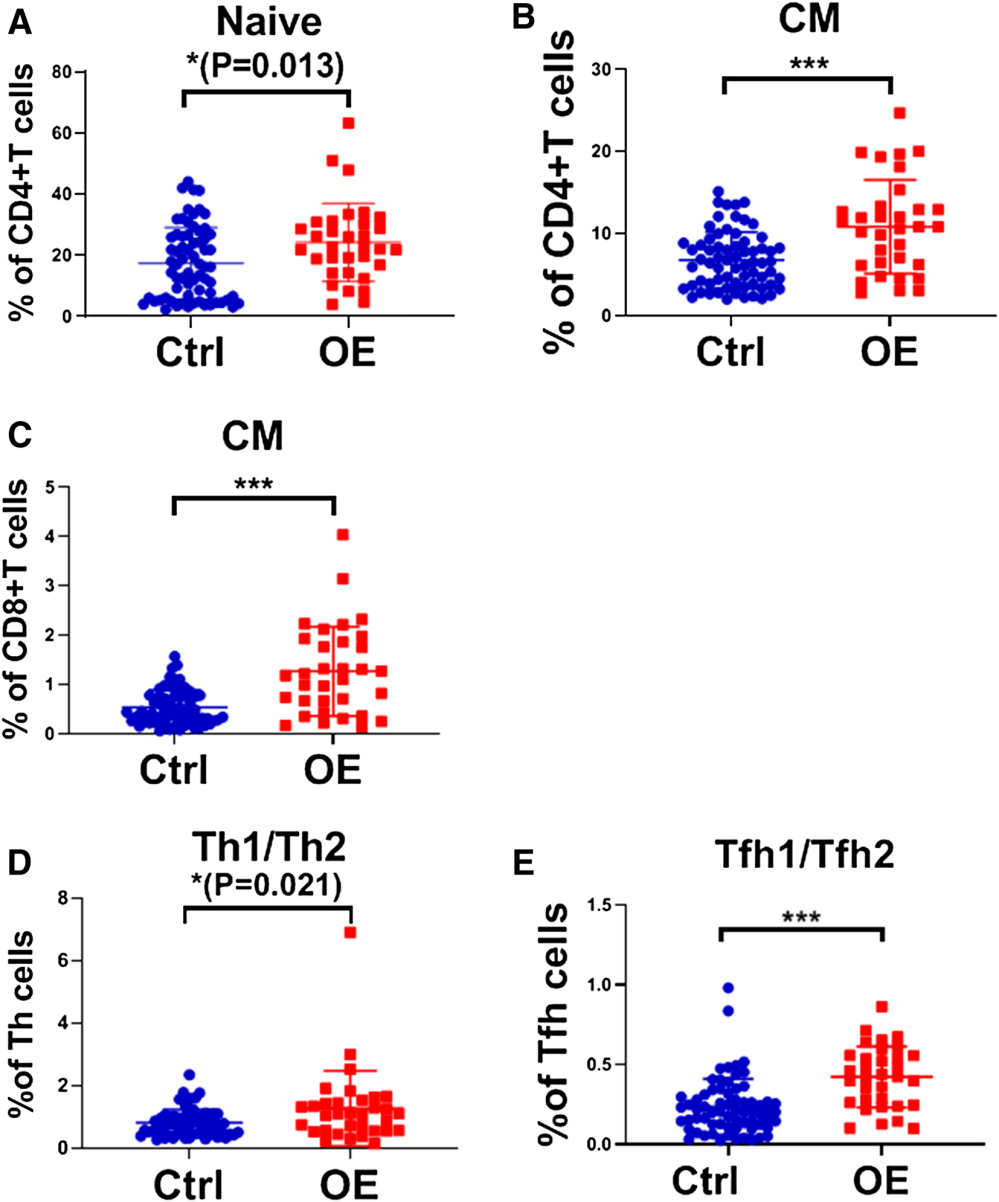
OE patients had higher levels of naïve CD4+ T cells, CM CD4+ T cells and CM CD8+ T cells, and higher ratios of Th1/Th2 and Tfh1/Tfh2, compared to healthy controls. (**A**) Percentage of naïve CD4+ T cells in CD4+ T cells, the gating strategy of naïve CD4+ T cells was ①+ ②+ ③+ (CD45RA+ /CCR7 +). (**B**) Percentage of CM CD4+ T cells in CD4+ T cells, CM CD4+ T cells gated by ①+ ②+ ③+ (CD45RA−/CCR7 +). (**C**) Percentage of CM CD8+ T cells in CD8+ T cells, CM CD8+ T cells gated by ①+ ②+ ④+ (CD45RA−/CCR7 +). (**D**) Th1/Th2, these two target cells were gated by ①+ ②+ ③+ ⑤+ (CXCR3+ /CCR4−) and ①+ ②+ ③+ ⑤+ (CXCR3−/CCR4 +), respectively. (**E**) Tfh1/Tfh2, these two target cells were gated by ①+ ②+ ③+ ⑥+ (CXCR3+ /CCR4−) and ①+ ②+ ③+ ⑥+ (CXCR3−/CCR4 +), respectively. The average values and their error bars were presented as median and interquartile range. Immunological characteristics between the patients and controls were compared by using Mann–Whitney U tests. * *P* < 0.05. **P < 0.01. *** *P* < 0.001. Ctrl: Control. OE: Ovarian endometrioma. ①, Lymphocyte. ②, CD3+. ③, CD4+. ④, CD8+. ⑤, CXCR5−. ⑥, CXCR5+.

**Figure 2:**
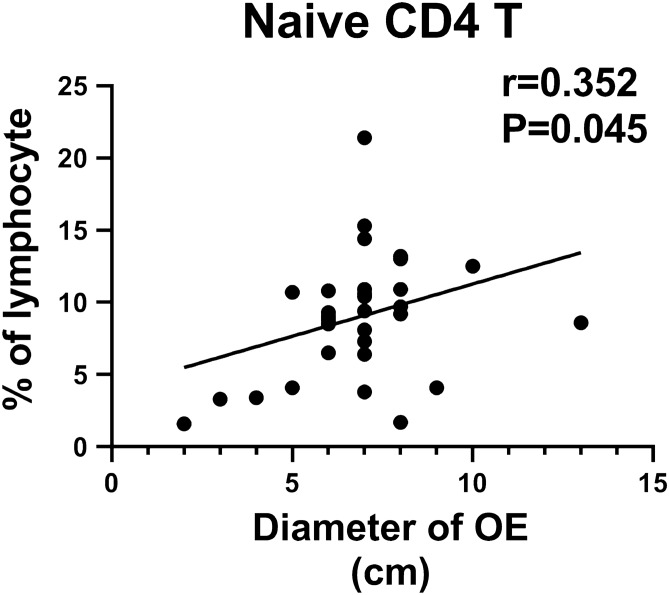
Naïve CD4+T cells was positively associated with patients’ OE size in OE. X axis is the diameter of OE. Y axis is the percentage of naïve CD4+T cells in lympocytes. The association was analyzed by a Spearman correlation analyse. OE: Ovarian endometrioma.

**Figure 3:**
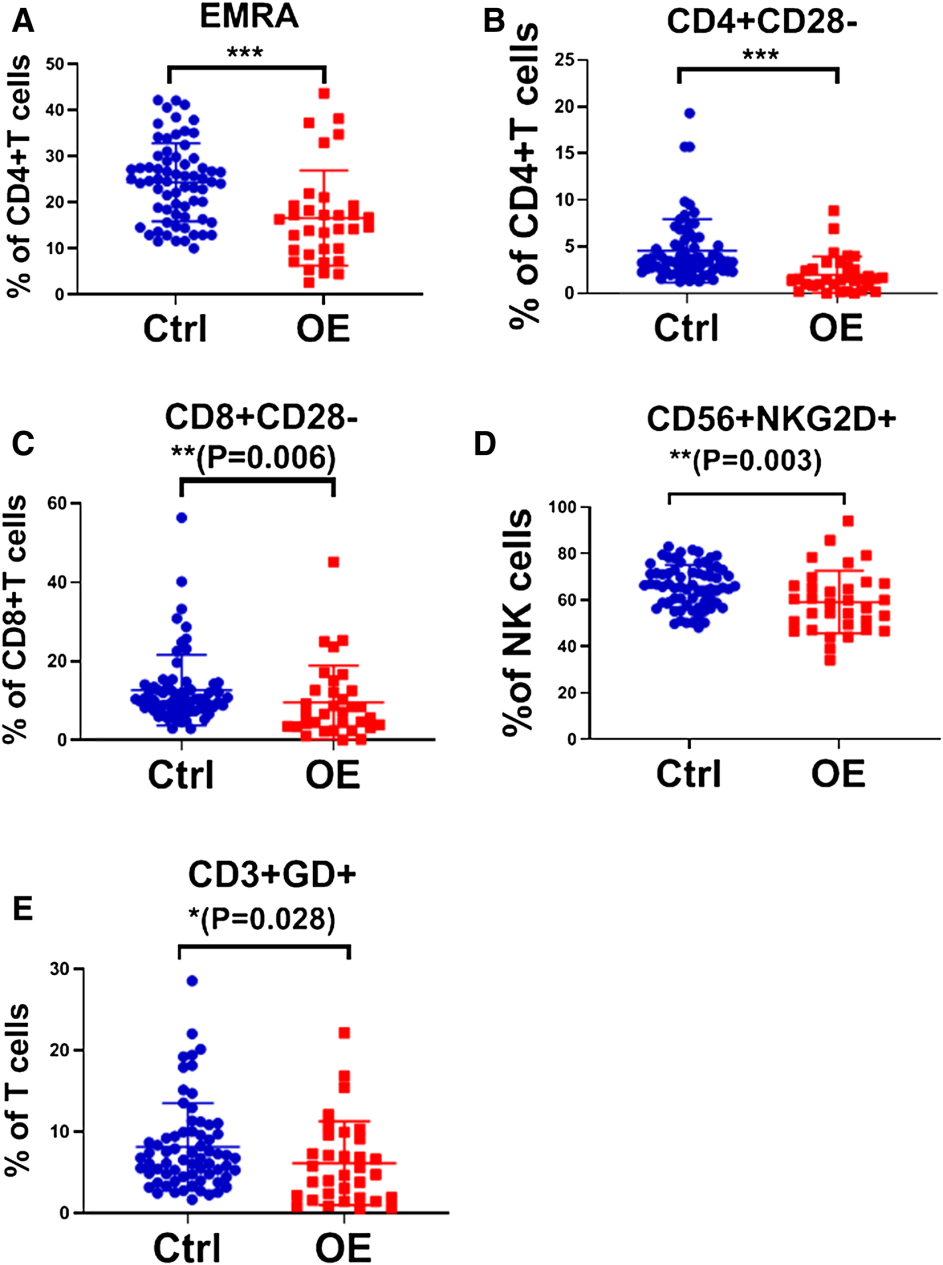
OE patients had lower levels of EMRA CD4+T cells, CD4+CD28− T cells, CD8+CD28− T cells, CD56+NKG2D+NK cells and γδ T cells, compared to healthy controls. (**A**) Percentage of EMRA CD4+T cells in CD4+T cells, the gating strategy of EMRA CD4+T cells was ①+②+③+(CD45RA+/CCR7−). (**B**) Percentage of CD4+CD28− in CD4+T cells, CD4+CD28− T cells gated by ①+②+③+(CD28−). (**C**) Percentage of CD8+CD28− T cells in CD8+T cells, CD8+CD28− T cells gated by ①+②+④+(CD28−). (**D**) Percentage of CD56+NKG2D+NK cells in lymphocytes, gated by ①+(CD3−)+(CD56 +)+(NKG2D +). (**E**) Percentage of γδ T cells in lymphocytes, gated by ①+②+(gammadelta +). Ctrl: Control. OE: Ovarian endometrioma. ①, Lymphocyte. ②, CD3+. ③, CD4+. ④, CD8+. The average values and their error bars were presented as median and interquartile range. Immunological characteristics between the patients and controls were compared by using Mann–Whitney U tests. * *P* < 0.05. **P < 0.01. *** *P* < 0.001.

**Figure 4:**
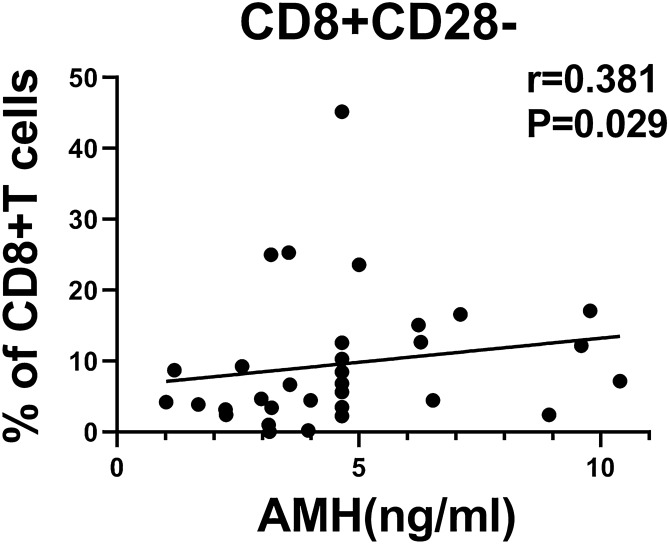
CD8+CD28− T cells was positively associated with the level of Anti-Mullerian hormone in OE patients. X axis is the level of Anti-Mullerian hormone. Y axis is percentage of CD8+CD28− T cells in CD8+T cells. The association was analyzed by a Spearman correlation analyse. OE: Ovarian endometrioma.

The baseline immune characteristics of OE patients were compared to those of the controls. The Mann–Whitney U tests indicated that the OE patients at baseline exhibited higher number of naïve CD4+ T cells, central memory (CM) CD4+ T cells and CM CD8+ T cells, and exhibited higher ratios of helper T cells (Th1/Th2) and follicular helper T cells (Tfh1/Tfh2) compared to those of the control subjects (all *P* values < 0.05) (Fig. 1). Moreover, the number of naïve CD4+ T cells was positively associated with the patient OE size (Spearman *r* = 0.352, *P* = 0.045) (Fig. 2). The patients usually exhibited lower levels of terminal differentiated effector memory (EMRA) CD4+ T cells, CD4+ CD28− T cells, CD8+ CD28− T cells, CD56+ NKG2D+ NK cells and γδ T cells (all *P* values < 0.05) (Fig. 3). In addition, a decreased number of CD8+ CD28− T cells was usually noted in patients with lower circulating anti-Mullerian hormone (Spearman *r* = 0.381, *P* = 0.029) (Fig. 4).

However, the percentage of total NK cells in lymphocytes was not significantly different between OE patients and control subjects (Suppl. Figure 1B). We also investigated the expression levels of the inhibitory receptor (KIR) and other activated receptors (NKP46 and NKP30) on NK cells. The results indicated that their expression levels did not reveal a significantly different distribution between cases and controls (all *P* values > 0.05, Suppl. Table 2).

We also performed *t* tests to compare the different distribution of immune cells and demonstrated that the majority of the results were similar to those derived from the Mann–Whitney U test, with the exception of CD8+ CD28− T, CD56+ NKP46+ NK and γδ T cells (Suppl. Table 2). Due to the large variations noted by the flow cytometry analyses and the apparent asymmetric distribution of CD8+ CD28− T cells and γδ T cells (Fig. 3), the results of the Mann–Whitney U test were slightly different from those of the *t* test. Based on the same reasons, Spearman correlation analyses, not Pearson correlation analyses, were performed to analyze the associations between immune characteristics and clinical phenotypes.

### The number of CD56+ NKG2D+ NK cells is significantly associated with postoperative fertility in OE patients

In the study period, 12 (36.4%) postoperative pregnancies occurred in OE cases and a total of 35 (51.5%) pregnancies occurred in healthy control subjects, as shown in Table 1. However, the rate and time of postoperative pregnancies were not significantly different between the two groups (both *P* values > 0.05).

Although the number of circulating lymphocytes was significantly lower in OE cases than that in the control subjects (Suppl. Figure 1A), it was not significantly different between the pregnant and the non-pregnant subjects either in the OE cases (Suppl. Figure 1C) or in the controls (Suppl. Figure 1D).

**Figure 5:**
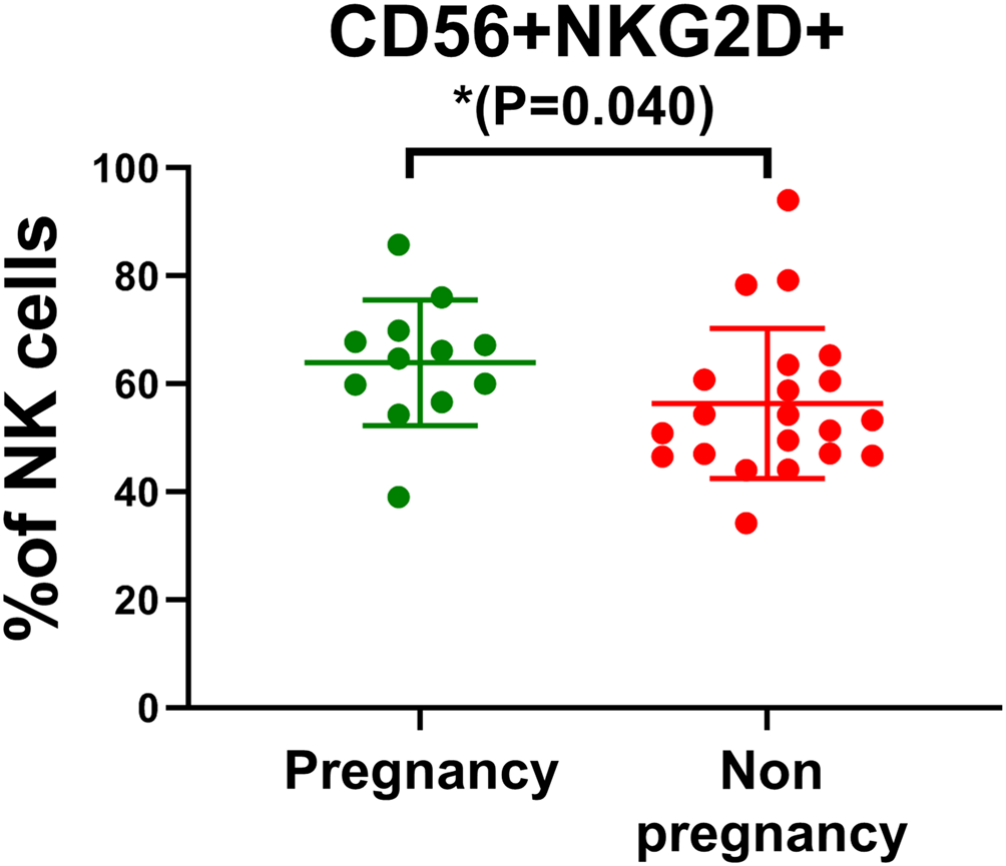
The percentage of CD56+NKG2D+cells in NK cells decreased in the patients without postoperative pregnancy compared to those with postoperative pregnancy. CD56+NKG2D+cells was gated by lymphocytes+(CD3−)+(CD56 +)+(NKG2D +). The average values and their error bars were presented as median and interquartile range. The difference between the patients and controls were compared by using Mann–Whitney U tests. * *P* < 0.05.

Among the statistically significant lymphocyte subsets between patients and controls, only the number of CD56+ NKG2D+ NK cells was significantly decreased in the patients without postoperative pregnancy compared with those pregnant subjects during follow-up (Mann–Whitney *U* test *z* = − 2.040, *P* = 0.040)(Fig. 5).

**Figure 6:**
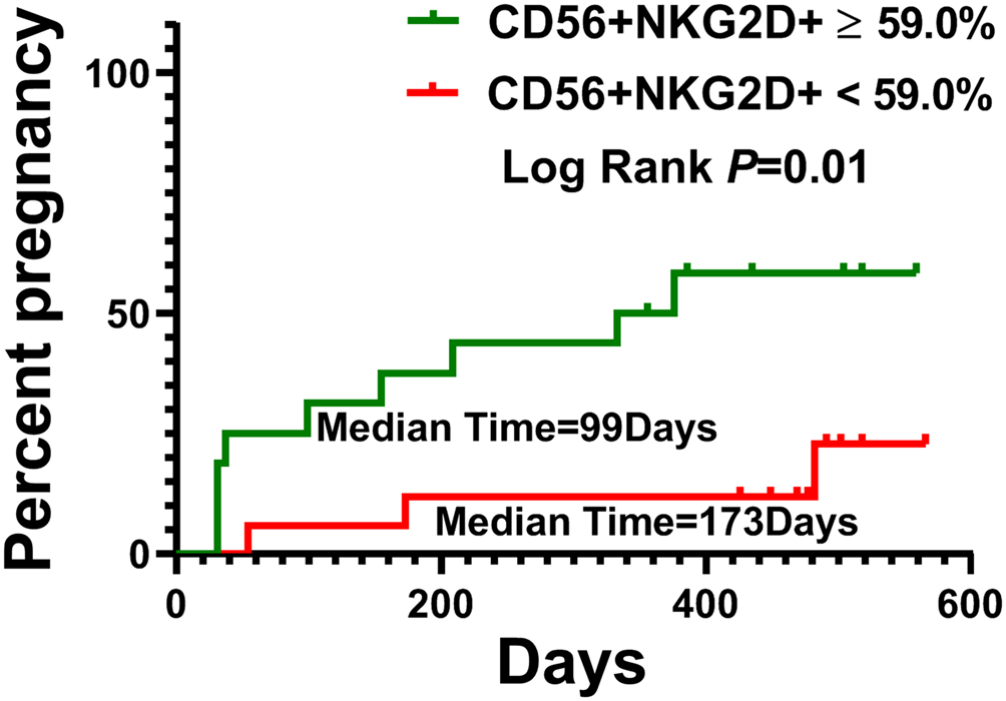
Kaplan–Meier survival analyses of postoperative fertility in OE patients by the level of circulating CD56+NKG2D+NK cells. Sixteen patients with the percentage of CD56+NKG2D+NK cells in total NK cells ≥ 59.0% (16 subjects: 9 with postoperative pregnancy, 7 censored); Seventeen patients with that percentage < 59.0% (17 subjects: 3 with postoperative pregnancy, 14 censored). Patients were censored at 31 December 2019.

Moreover, the number of CD56+ NKG2D+ NK cell was a significant predictor of postoperative fertility in three different COX regression models. As shown in Table 2, Model I was used to analyze the crude hazard ratio (HR) and its 95% confidence interval (CI); Model II was used to adjust certain reported important confounding factors in infertility of OE, including age^22^, OE size^23^ and circulating anti-Mullerian hormone^24^; model III was used to further adjust additional pathogenic and prognostic factors of OE, including body mass index, age at menarche, OE location and surgical approach^23,25,26^. Following adjustment of all these confounding factors, the immune index was associated with an apparent drop in postoperative pregnancy rate (adjusted HR = 0.127, 95% CI = 0.024–0.675, *P* = 0.015). However, these reported risk factors were not significant in the multiple COX model (Suppl. Table 3). Furthermore, the lower number of circulating CD56+ NKG2D+ NK cells (< 59%, the median) was associated with a significant delay to successful pregnancy (median time, 173 vs. 99 days, log-rank *P* = 0.013) (Table 2 and Fig. 6). However, we did not find significant associations between other immune cell subpopulations and the incidence of postoperative pregnancy in the present study.

**Table 2:** Analysis of the percentage of CD56+ NKG2D+ cells and postoperative fertility in ovarian endometrioma.

CD56+ NKG2D+ (%)	Pregnancy/cases (n)	MPT (days)	Log-Rank *P* value	Cox regression					
				Model I^*a*^		Model II^*b*^		Model III^*c*^	
				HR (95% *CI*)	*P*	HR (95% *CI*)	*P*	HR (95% *CI*)	*P*
≥ 59.0	9/16	173		1.00 (ref.)		1.00 (ref.)		1.00 (ref.)	
< 59.0	3/17	99	0.013	0.220(0.059–0.822)	0.024	0.240(0.063–0.915)	0.037	0.127(0.024–0.675)	0.015

Notes: MPT, median pregnancy time; HR, hazard ratio; CI, confidence interval; ^*a*^ no adjustment; ^*b*^ adjusting age, OE size, and circulating anti-Mullerian hormone; ^*c*^ adjusting adjusting the confounding factors including age, body mass index, age at menarche, location and size of OE, surgical approach, and circulating anti-Mullerian hormone.

In contrast to these observations, the number of CD56+ NKG2D+ NK cells in the control subjects did not exhibit a significantly different distribution between the number of pregnancies and non-pregnancies (Suppl. Figure 1E). Moreover, the number of circulating CD56+ NKG2D+ NK cells in the control subjects was not significantly associated with the fertility rate as determined in the three COX regression models (all *P* values > 0.05, Suppl. Table 4). In addition, it was not significantly associated with the time to pregnancy in the control subjects (median time, 126 vs. 99 days, log-rank *P* = 0.845).

## Discussion

In the present study, we found that the number of circulating CD56+ NKG2D+ NK cells was decreased in OE patients; moreover, the lower levels of CD56+ NKG2D+ NK cells were associated with a poor postoperative pregnancy rate and a delayed time to successful pregnancy in OE patients. However, the number of CD56+ NKG2D+ NK cells in the control subjects was not significantly associated with the rate and time for pregnancy during the study period. To the best of our knowledge, the present study was the first to specifically examine the influence of circulating CD56+ NKG2D+ NK cells as a prognostic factor of postoperative fertility in patients with OE.

Several studies have reported an impaired NK cell function in endometriosis patients. However, a limited number of studies have specifically examined the influence of circulating CD56+ NKG2D+ NK cells as a prognostic factor of postoperative fertility in patients with OE. For example, the expression levels of NKG2D on CD56+ NK cells in the peritoneal fluid of endometriosis patients are usually lower than those of healthy subjects^27^. NK cell cytotoxicity in the serum and in peritoneal fluid is both decreased in endometriosis patients^28^. In addition, uterine NK progenitor cell populations in endometriosis patients are markedly lower in subjects who proceeded with failed embryo implantations compared to those with successful implantation^18^. Fukui et al. suggested that the abnormal of uterine and/or peripheral blood NK cells was highly associated with reproductive failure, including the patients with pelvic endometriosis^29^. The findings on the association between CD56+ NKG2D+ NK cell and postoperative fertility in OE patients were consistent with the aforementioned reports.

NKG2D is an important activated receptor, which is mainly expressed in NK cells and can trigger their cytotoxic response^30^. The decrease in the expression of NKG2D and the increase in the levels of certain inhibitory receptors expressed on NK cells can damage their function and cause immune escape, which makes the peritoneal infiltration and proliferation of ectopic endometrial cells easier and consequently increases the incidence of OE^31^. In addition, ectopic endometrial stromal cells can hamper NK cell differentiation and impair their cytotoxic function, thus promoting the development of OE^32^. Notably, the expression of NKG2D in uterine NK cells is significantly downregulated in endometriosis patients and is induced by endometrial stromal cells and uterine macrophages. This can further trigger the immune escape of ectopic fragments and promote the occurrence and the development of OE^33^. In addition to their role in the etiology of OE, NK cells can further regulate reproductive process, which can increase the adaptation of the mother to pregnancy and regulate placental development and angiogenesis^16^. For example, Marlin et al. demonstrated significantly increased expression of the NKG2D receptor at the decidual NK cell surface during pregnancy, whereas the expression levels of other activator receptors, such as NKP30 and NKP44 were apparently decreased^34^. Therefore, the findings on the effect of circulating CD56+ NKG2D+ NK cells on postoperative fertility in OE patients are biologically plausible.

The present study contains certain advantages. The number of peripheral CD56+ NKG2D+ NK cells can be used as a non-invasive biomarker for postoperative fertility in OE. It is easily tested prior to operation and can be used to optimize the choice of curative methods. It has been proposed that peripheral NK subsets cannot precisely represent the local immune status in the uterus. However, OE is an inflammatory disease, which involves multiple organs, including the ovary and the uterus^12^. The peripheral subsets of immune cells are also considered suitable for evaluating the inflammatory status of OE^13,14^. Therefore, the number of peripheral CD56+ NKG2D+ NK cells can be used as a preoperative biomarker in OE patients.

The present study contains certain limitations, which are highlighted as follows: 1) A limited sample size was used in the present study. However, we found that CD56+ NKG2D+ NK cells were the only significant lymphocyte subpopulation associated with postoperative fertility in OE patients. Moreover, it was the only significant predictor for postoperative fertility as determined by the multiple COX regression model including various clinical characteristics. Following adjustment of specific confounding factors, including age, body mass index, age at menarche, location and size of OE, surgical approach and levels of circulating anti-Mullerian hormone, the number of CD56+ NKG2D+ NK cells was still significantly associated with a poor rate of postoperative fertility. Furthermore, Kaplan–Meier survival analyses indicated that it was associated with an apparent delay to successful pregnancy in OE patients. These results suggested that CD56+ NKG2D+ NK cells could be used as a candidate biomarker for postoperative fertility in OE. 2) Only one blood profiling analysis was used in the present study. Although the present study examined the use of noninvasive biomarkers prior to the operation, the subsequent effects caused on these immune indices following surgery are also critical. 3) The present study analyzed only specific lymphocyte subsets. A larger immune profiling panel including assessment of the levels of serum cytokines is warranted in future studies. 4) Due to the small sample size of the present study, several epidemiological variables may provide potential bias to the study results, in a way that the frequency of sexual intercourse among these two groups following the surgery affects the outcomes of the analysis. However, all these OE patients arrived at our hospital to improve fertility by operation, implying that they will make the necessary preparation in order to conceive after operation. In addition, the median follow-up period of these cases was 435 days (not significantly different to that of the control subjects), which is long enough to identify the difference in postoperative fertility between these groups of higher and lower CD56+ NKG2D+ NK cell number. Taken together, larger population studies from different ethnic population, including a larger immune profiling panel and a more detailed questionnaire with epidemiological factors, should be performed to validate the conclusions of our study.

In conclusion, the present findings suggested that a lower number of circulating CD56+ NKG2D+ NK cells can predict a poor postoperative fertility outcome in OE subjects. Larger population studies are warranted.

## Methods

### Subjects

Improving fertility is one of the major reasons for operation in OE. According to Guideline for the diagnosis and treatment of endometriosis of Chinese Society of Obstetrics and Gynecology (CSOG)^35^ and American College of Obstetricians and Gynecologists (ACOG)^36^, patients’ age, endometriosis fertility index, childbearing desire and other factors should be considered before operation. Therefore, only the OE patients with fertility desire and those should be treated by laparoscope and/or hysteroscope were recruited in this study. The present cohort study was carried out between June 2018 and January 2019. Its flow diagram is described in Suppl. Figure 2. Briefly, after examined according to Guideline of CSOG^35^, all the available OE patients aged 20 ~ 40 years who arrived at our hospital for improving fertility by operation and those willing to participate in this study with a response rate > 95%, were recruited. The diagnoses of OE were performed by B-ultrasonic examination and 50 OE patients were recruited. Based on CSOG^35^ and ACOG^36^, the husbands of these patients were all examined for sterility assessment. No male subjects with infertility were noted in the present study. All OE patients were treated by laparoscopy and/or hysteroscopy according to our hospital routine method. Following exclusion of 9 patients due to co-morbidity with uterine leyomyoma and 8 patients with wrong diagnoses verified by pathological examination, 33 patients were further studied. The day prior to the operation, a 3 ml anticlotting blood sample was collected for immune tests from each patient following submission of signed written informed consent form. The patient information on age, height, weight, the history of menses and reproduction and the clinical characteristics of OE were recorded from their medical records.

To compare the baseline characteristics of the patients, a total of 72 healthy females (controls) aged between 20 and 40 years were recruited by random sampling from the subjects who arrived at our hospital for pre-pregnancy medical examinations from September to November 2018. All subjects that arrived on Saturday were recruited with a response rate > 90%. The healthy status of these subjects was re-assessed by a series of pre-pregnancy medical examinations prior to December 2019. The subjects with ovarian and uterine diseases were excluded according to B-ultrasonic examinations at pre-pregnancy medical examinations. Following exclusion of 4 patients with recurrent abortions, 68 controls were included in the present study. The blood samples of the control samples were also collected with their informed permission on the day of pre-pregnancy medical examination. In addition, the information on age, height, weight and the history of menses and reproduction of the controls was recorded from their medical records. Due to ethnic reasons, the information on circulating anti-Mullerian hormone and Cancer antigen 125 was not collected in the control subjects.

The present study was conducted according to The Code of Ethics of the World Medical Association (Declaration of Helsinki) and was approved by the Ethics Committee of Shenzhen Baoan Mothers’ and Children’s Hospital, Jinan University (IRB: LLSC-2018-08-04-01).

### Flow cytometry analyses

Peripheral lymphocyte subpopulations of the patients and control subjects were measured by flow cytometry according to our routine methods^37^. Briefly, following addition of the FACS lysing solution (BD Biosciences, San Jose, CA, USA) hemolysis was performed to separate peripheral white blood cells. Phosphate-buffered saline was used to wash the precipitates twice and the cells were labeled by the following antibodies: APC-H7-conjugated anti-CD4; PerCP-Cy5.5-conjugated anti-CD3; BV510-conjugated anti-CD8, anti-CD196 and anti-NKP46; FITC-conjugated anti-CD45RA; Alexa Fluor 647-conjugated anti-CCR7, anti-NKP30 and anti-CXCR5; PE-conjugated anti-CD279 and anti-Vδ2; PE-Cy7-conjugated anti-CD28 and anti-NKG2D; Alexa Fluor 488-conjugated anti-CD183, and BB515-conjugated anti-PD-1; BV421-conjugated anti-CD194. The antibodies were purchased from BD Biosciences.

All the samples were tested on a BD LSR Fortessa cell analyzer (BD Biosciences) at Shuangzhi Purui Medical Laboratory Co., Ltd. (China). The FlowJo 10.1 software (Tree Star Inc., Ashland, USA) was used to analyze the data. To avoid bias caused in the flow cytometry analysis, all examinations were performed following the adjustment and calibration of the instrument according to the manual. The immune index of each sample was analyzed by gating as described in our previous report^37^. The definition, markers and the gating strategy of various lymphocyte subsets were described as Suppl. Table 5. Original analyses were performed by the Shuangzhi Purui Medical Laboratory Co. However, all results were re-assessed by one investigator who participated in the study (M.L.).

### Operation and analysis of postoperative fertility

All OE patients were treated by laparoscopy and/or hysteroscopy according to our hospital routine methods and were discharged from our hospital in a week. All these cases were trying to conceive. All cases and control subjects were followed up by a series of pre-pregnancy medical examinations before December 2019. They were followed up to the date of pregnancy or were censored on December 31 2019. The pregnancy and its date were determined by the test of human chorionic gonadotropin, B-ultrasonic examination and the last menstrual period. Subsequently, the association between immune characteristics and postoperative fertility in these patients was analyzed using multiple Cox regression analyses and Kaplan–Meier survival analyses.

### Statistical analysis

To avoid bias of data collection, the data used for analyses were double-checked from the original medical records by two different research assistants. The groupings of variables were set by the median value. Missing data are very common in clinical research. Although imputed data are not actual data, they are verified as constructed values that increase the sensitivity of testing and are broadly used in clinical and epidemiological research^38,39^. Missing data were observed in the present study, yet to a considerably low percentage (< 10% of 6 variables in OE patients, Table 1) and were replaced by the median value. The analysis results without the imputed data were similar to those with the imputed data in the present study (Suppl. Table 6).

The baseline clinical and immunological characteristics between the patients and control subjects were compared by the Mann–Whitney U and the *t* tests, and the associations between immune indices and baseline clinical phenotypes were performed in 33 OE patients by Spearman correlation analyses. The Mann–Whitney U test was also used to compare the different immune indices between the postoperative pregnancies and the subjects without pregnancies following operation. The association between immune indices and postoperative pregnancy was analyzed by multiple Cox regression (forward model) and Kaplan–Meier survival analyses. To avoid the false-positive report of the association of immune indices with postoperative fertility, we analyzed initially the most significant immune indices between the postoperative pregnancies and the non-pregnancy cases using the Mann–Whitney U tests; subsequently the association between the most significant immune indices and postoperative pregnancy was analyzed using Kaplan–Meier survival analyses and multiple Cox regression analyses following adjustment of possible confounding factors. Statistical analyses were conducted using the SPSS 25.0 software (Armonk, NY, IBM Corp, USA) and the results were interpreted at the 5% level of significance. The relevant graphs were constructed by GraphPad Prism8.0.2 (GraphPad Software, USA).

## Supplementary information

Supplementary Information 1.

Supplementary Information 2.

Supplementary Information 3.

## Data Availability

Original data is available on the journal web (Suppl. Table 1).
