# Supplementary material for: Circulating CD56+ NKG2D+ NK cells and postoperative fertility in ovarian endometrioma
Source: Sci Rep. 2020 Oct 29;10:18598. doi: 10.1038/s41598-020-75570-z (PMC7596045; doi:10.1038/s41598-020-75570-z)
Supplement: Supplementary file 1 — Supplementary Information 1. [file 41598_2020_75570_MOESM1_ESM.pdf]

Circulating CD56+NKG2D+ NK cells and postoperative fertility in ovarian endometrioma

Authors: Zhi-Qin Liu,[1#] Mei-Yin Lu,[2#] Bin Liu,[2*]

# These two authors contributed equally to this article.

Affiliations:

1 Department of Obstetrics and Gynecology, Shenzhen Baoan Mothers' and Children's Hospital, Jinan University, Shenzhen 518102, Guangdong, China

2 Department of Biobank, Shenzhen Baoan Mothers' and Children's Hospital, Jinan University, Shenzhen 518102, Guangdong, China

Correspondence

Correspondence should be addressed to Bin Liu, PhD, MD, Department of Biobank, Shenzhen Baoan Mothers' and Children's Hospital, Jinan University, Shenzhen 518102, Guangdong, China

E-mail: gz12liubin@163.com

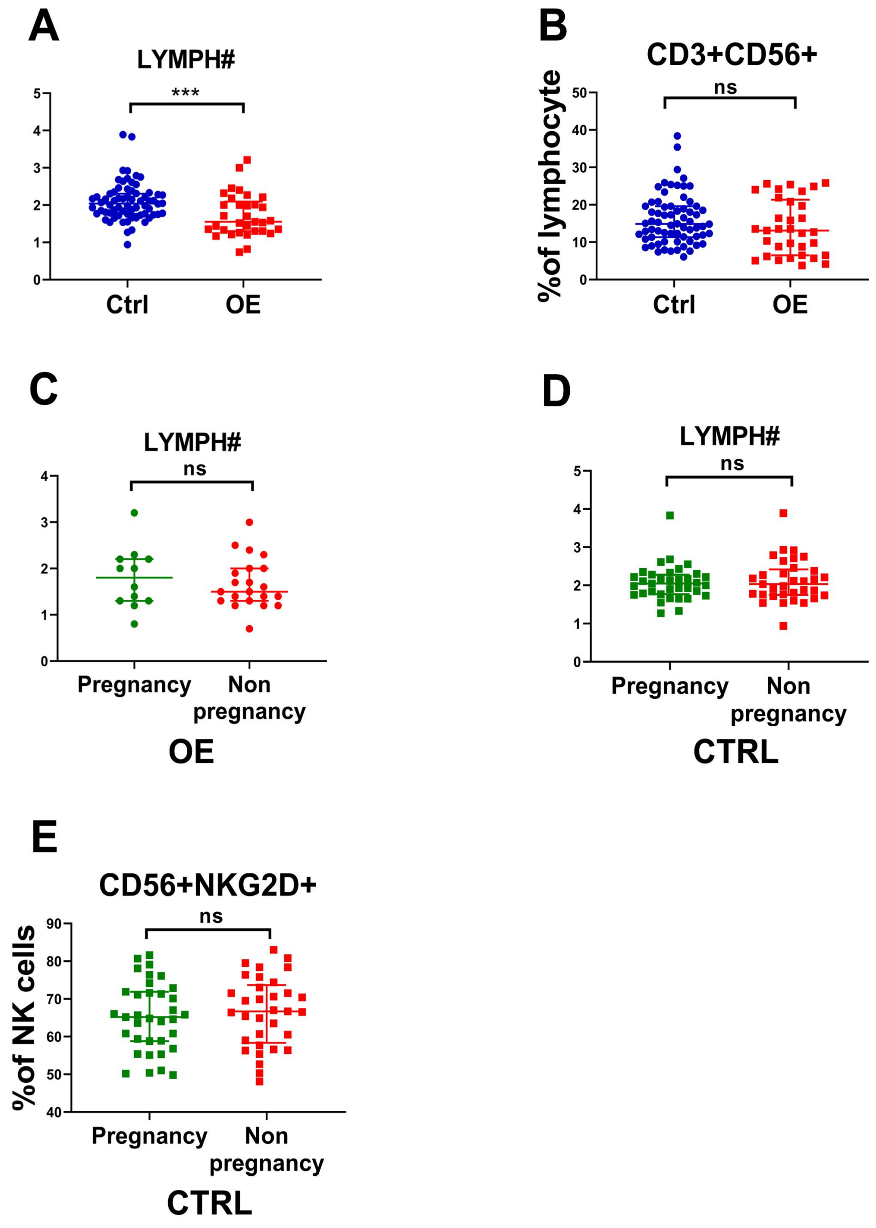

Suppl. Figure 1. Comparision of distribution of lymphocytes and NK cells in OE patients and controls, in postoperative preganancies and non-preganancies. **(A)**, circulating lymphocytes in OE patients and controls. **(B)**, percentage of NK cells in OE patients and controls, gated by lymphocytes+(CD3-)+(CD56+). **(C)**, circulating lymphocytes in postoperative pregnancies and non-preganancies of the OE patients. **(D)**, circulating lymphocytes in postoperative pregnancies and non-preganancies of the controls. **(E)**, percentage of CD56+NKG2D+ NK cells in NK cells in in postoperative pregnancies and non-preganancies of the controls. The average values and their error bars were presented as median and interquartile range. Immunological characteristics between the patients and controls were compared by using Mann-Whitney U tests. ns, non-significant. *** $P < 0.001$. OE: ovarian endometrioma. CTRL: control.

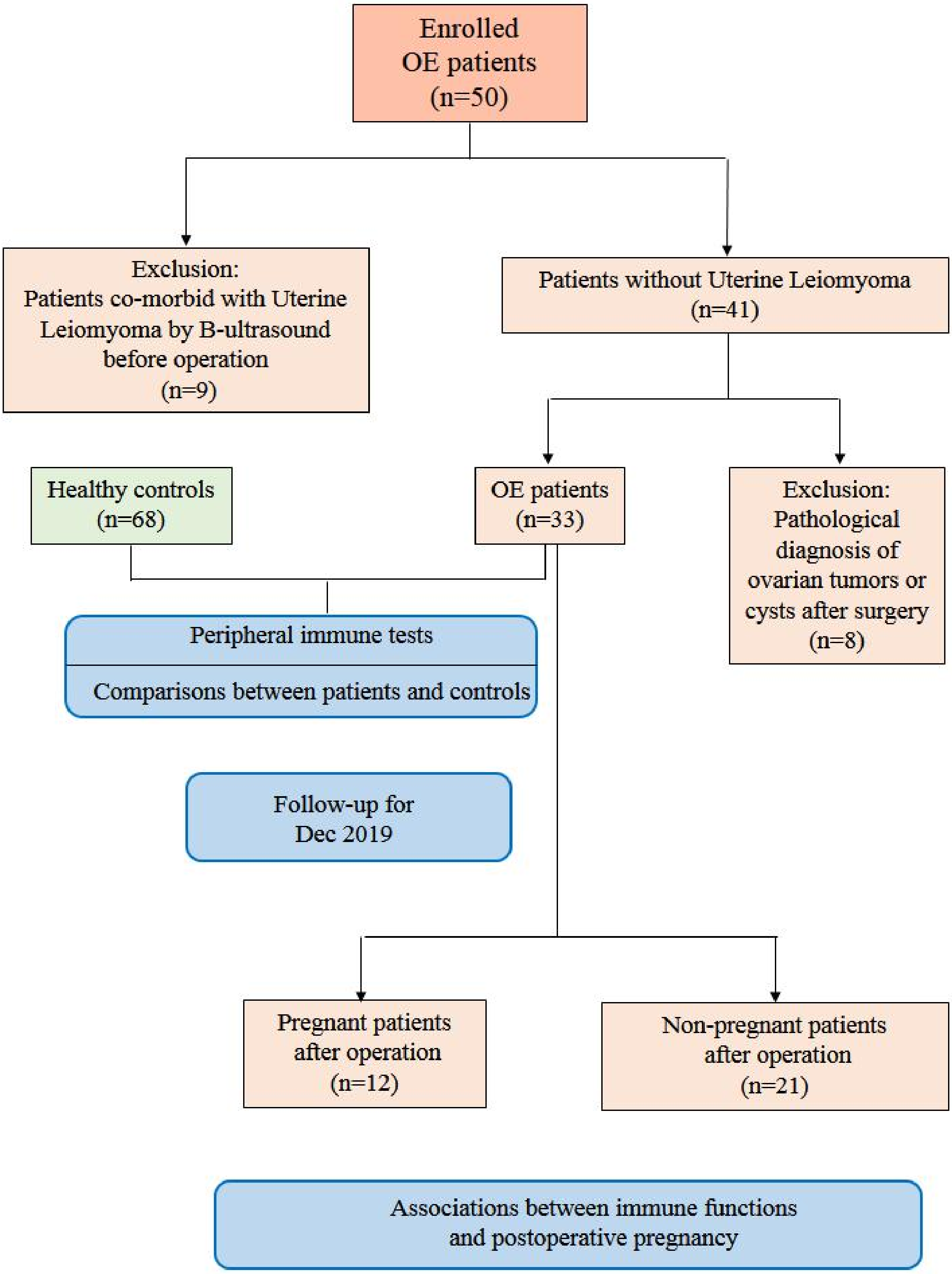

Suppl. Figure 2. The flow diagram of this study. OE: ovarian endometrioma.
