# Supplementary material for: Circulating CD56+ NKG2D+ NK cells and postoperative fertility in ovarian endometrioma
Source: Sci Rep. 2020 Oct 29;10:18598. doi: 10.1038/s41598-020-75570-z (PMC7596045; doi:10.1038/s41598-020-75570-z)
Supplement: Supplementary file 2 — Supplementary Information 2. [file 41598_2020_75570_MOESM2_ESM.pdf]

Title: Circulating CD56+NKG2D+ NK cells and postoperative fertility in ovarian endometriom

Authors: Zhi-Qin Liu,1# Mei-Yin Lu,2# Bin Liu,2*

Suppl. Table 1. Original data of 33 cases with ovarian endometrioma

Serial number	CD56+NKG2D+ (% of NK cells)	CD56+NKP30+ (% of NK cells)	CD56+NKP46+ (% of NK cells)	CD56+ KIR+ (% of NK cells)	Age (Years)
1	59.9	23.6	28.5	9.8	29
2	53.3	89.7	87.3	29.4	32
3	54.3	82.1	87.1	11.4	29
4	60.5	69.0	89.7	6.2	30
5	60.0	85.2	87.7	8.9	28
6	56.6	88.8	92.7	9.6	26
7	46.5	51.3	76.2	5.8	39
8	65.3	48.9	64.5	11.4	25
9	67.8	63.4	55.4	7.4	25
10	60.8	62.6	88.7	5.4	28
11	44.0	39.0	42.6	5.5	32
12	54.4	45.2	46.0	5.9	18
13	54.3	35.7	50.9	37.2	29
14	47.0	38.5	50.2	11.0	36
15	34.2	72.5	78.2	15.1	36
16	64.7	39.4	64.4	2.4	27
17	49.5	75.9	88.5	6.0	32
18	51.4	69.4	93.6	4.7	40
19	47.1	60.6	88.9	6.0	26
20	50.9	66.3	86.6	4.9	40
21	76.0	80.3	76.6	5.7	26
22	46.7	15.4	36.5	5.4	26
23	39.0	41.5	52.2	24.9	35
24	63.5	71.5	84.5	16.7	37
25	67.2	57.5	84.5	12.6	30
26	44.1	23.6	35.8	5.9	33
27	58.8	27.2	46.5	2.5	33
28	69.9	78.1	79.0	10.3	30
29	85.8	67.8	64.6	7.7	32
30	66.1	68.5	57.1	8.2	26
31	94.0	95.6	53.2	5.0	28
32	78.4	74.9	85.3	17.2	27
33	79.2	64.2	59.8	5.8	35

ıa

BMI (Kg/m2)	Age of menarche (Years)	Menstrual cycle (Days)	Menstrual duration (Days)	Dysmenorrhea	Position of OE	Diameter of OE (cm)
21.8	17	45	4	Yes	Right	10
17.6	14	30	6	No	Left	5
17.7	13	35	7	Yes	Left	8
16.4	15	30	8	Yes	Right	9
18.8	14	30	4	Yes	Right	6
21.0	15	30	7	Yes	Left	4
17.5	14	30	7	No	Left	13
18.1	15	30	5	Yes	Bilateral	6
17.8	14	25	7	Yes	Left	8
19.2	11	30	5	Yes	Left	8
19.1	14	30	7	No	Left	7
24.4	12	30	7	Yes	Left	6
20.3	12	30	5	No	Left	8
19.9	13	30	5	No	Left	7
20.3	14	30	7	No	Left	7
20.5	13	34	5	Yes	Right	6
19.1	14	30	7	No	Right	7
22.9	14	30	7	No	Right	7
24.5	14	30	7	No	Bilateral	7
19.6	16	28	7	No	Left	7
23.9	13	50	3	Yes	Right	8
19.3	13	31	5	Yes	Right	3
21.0	13	31	5	No	Left	2
23.2	15	35	4	No	Left	7
20.8	15	35	7	Yes	Bilateral	7
18.8	12	30	7	Yes	Bilateral	8
22.8	14	30	5	No	Right	6
20.1	13	30	7	Yes	Left	7
20.0	13	30	5	No	Right	6
20.9	13	30	5	No	Left	6
19.8	14	30	7	No	Bilateral	7
19.5	15	30	7	No	Right	7
20.8	15	30	5	Yes	Bilateral	5

Complication	Surgical approach	Operation time	Endtime of follow-up	Follow-up days	Pregnancy or not after operation
Pelvic endometriosis	Laparoscope	13-Jun-18	14-Jul-18	31	Yes
Deep infiltrating endometriosis	Laparoscope	13-Jun-18	31-Dec-19	566	No
Pelvic endometriosis	Laparoscope	20-Jun-18	15-Oct-19	482	Yes
Deep infiltrating endometriosis	Laparoscope	20-Jun-18	31-Dec-19	559	No
Pelvic endometriosis	Laparoscope& hysteroscope	4-Jul-18	5-Aug-18	31	Yes
Pelvic endometriosis	Laparoscope& hysteroscope	4-Jul-18	27-Aug-18	54	Yes
-	Laparoscope	31-Jul-18	31-Dec-19	518	No
Pelvic endometriosis	Laparoscope& hysteroscope	31-Jul-18	31-Dec-19	518	No
-	Laparoscope	6-Aug-18	12-Sep-18	36	Yes
-	Laparoscope	14-Aug-18	31-Dec-19	504	No
Pelvic endometriosis	Laparoscope	27-Aug-18	31-Dec-19	491	No
Pelvic endometriosis	Laparoscope	15-Aug-18	31-Dec-19	503	No
Pelvic endometriosis	Laparoscope	16-Aug-18	31-Dec-19	502	No
-	Laparoscope	16-Aug-18	31-Dec-19	502	No
Pelvic endometriosis	Laparoscope	16-Aug-18	31-Dec-19	502	No
-	Laparoscope& hysteroscope	22-Aug-18	24-Jan-19	155	Yes
Pelvic endometriosis	Laparoscope	10-Sep-18	31-Dec-19	477	No
Pelvic endometriosis	Laparoscope& hysteroscope	10-Sep-18	31-Dec-19	477	No
Deep infiltrating endometriosis	Laparoscope& hysteroscope	10-Sep-18	31-Dec-19	477	No
Pelvic endometriosis	Laparoscope	18-Sep-18	31-Dec-19	469	No
-	Laparoscope	25-Sep-18	24-Aug-19	333	Yes
-	Laparoscope& hysteroscope	8-Oct-18	31-Dec-19	449	No
Pelvic endometriosis	Hysteroscope	8-Oct-18	30-Mar-19	173	Yes
Pelvic endometriosis	Laparoscope	22-Oct-18	31-Dec-19	435	No
Pelvic endometriosis	Laparoscope	22-Oct-18	29-Jan-19	99	Yes
Deep infiltrating endometriosis	Laparoscope	30-Oct-18	31-Dec-19	427	No
Pelvic endometriosis	Laparoscope	31-Oct-18	31-Dec-19	426	No
Pelvic endometriosis	Laparoscope& hysteroscope	7-Nov-18	4-Jun-19	209	Yes
Pelvic endometriosis	Laparoscope	21-Nov-18	2-Dec-19	376	Yes
-	Hysteroscope	18-Dec-18	19-Jan-19	31	Yes
Deep infiltrating endometriosis	Laparoscope	10-Dec-18	31-Dec-19	386	No
-	Laparoscope& hysteroscope	9-Jan-19	31-Dec-19	356	No
Deep infiltrating endometriosis	Laparoscope& hysteroscope	9-Jan-19	31-Dec-19	356	No

Pregnancy time	WBC (10^9/L)	Hb (10^12/L)	RBC (10^12/L)	AMH (ng/l)
14-Jul-18	7.95	124	5.38	4.00
	3.49	125	4.25	4.65
15-Oct-19	6.48	138	4.56	8.93
	4.74	125	4.25	3.13
5-Aug-18	7.74	118	4.16	4.65
27-Aug-18	4.33	122	4.53	7.10
	6.35	108	5.59	2.24
	8.36	106	4.15	3.19
12-Sep-18	4.95	136	4.27	6.23
	4.66	147	4.87	1.68
	6.12	126	4.57	4.65
	6.86	123	4.61	9.60
	9.39	126	5.94	3.57
	5.93	120	4.19	2.58
	6.12	126	4.57	4.65
24-Jan-19	4.86	107	3.86	3.55
	6.12	126	4.57	4.65
	6.12	126	4.57	4.65
	6.12	126	4.57	4.65
	4.11	111	4.29	2.98
24-Aug-19	11.20	153	4.48	6.53
	6.50	114	5.98	1.01
30-Mar-19	8.56	127	4.18	1.18
	5.85	146	4.44	4.65
29-Jan-19	5.16	134	3.95	10.40
	4.61	120	4.03	4.65
	5.46	126	4.09	2.25
4-Jun-19	5.04	126	4.43	3.95
2-Dec-19	3.46	126	4.42	3.15
19-Jan-19	7.02	140	4.75	6.28
	5.17	135	4.4	9.78
	6.90	123	4.28	5.00
	6.10	109	5.59	3.18
