# Supplementary material for: Circulating CD56+ NKG2D+ NK cells and postoperative fertility in ovarian endometrioma
Source: Sci Rep. 2020 Oct 29;10:18598. doi: 10.1038/s41598-020-75570-z (PMC7596045; doi:10.1038/s41598-020-75570-z)
Supplement: Supplementary file 3 — Supplementary Information 3. [file 41598_2020_75570_MOESM3_ESM.pdf]

Circulating CD56+NKG2D+ NK cells and postoperative fertility in ovarian endometrioma

Authors: Zhi-Qin Liu,[1#] Mei-Yin Lu,[2#] Bin Liu,[2*]

# These two authors contributed equally to this article.

Affiliations:

1 Department of Obstetrics and Gynecology, Shenzhen Baoan Mothers' and Children's Hospital, Jinan University, Shenzhen 518102, Guangdong, China

2 Department of Biobank, Shenzhen Baoan Mothers' and Children's Hospital, Jinan University, Shenzhen 518102, Guangdong, China

Correspondence

Correspondence should be addressed to Bin Liu, PhD, MD, Department of Biobank, Shenzhen Baoan Mothers' and Children's Hospital, Jinan University, Shenzhen 518102, Guangdong, China

E-mail: gz12liubin@163.com

Suppl. Table 2. Comparison of lymphocyte subpopulations between 33 OE patients and 68 controls (Mann-Whitney U-test and T-test).

	P **values for Mann-Whitney U-test**	***P*** **values for** ***t*** **test**
Naïve CD4+ T cells	0.013	0.009
CM CD4+ T cells	<0.001	<0.001
EMRA CD4+ T cells	<0.001	<0.001
CD4+ CD28- T cells	<0.001	<0.001
CM CD8+ T cells	<0.001	<0.001
CD8+ CD28- T cells	0.006	0.114
Th1/Th2	0.021	0.042
Tfh1/Tfh2	<0.001	<0.001
CD56+ NKG2D+ NK cells	0.003	0.012
CD56+ NKP30+ NK cells	0.891	0.945
CD56+ NKP46+ NK cells	0.092	0.021
CD56+ KIR+ NK cells	0.487	0.558
γδ T cells	0.028	0.077

Suppl. Table 3. Group variables of multiple Cox regression in cases in the model III of Table 2.

**Variables**	**Cases (n)**	**Hazard ratio**	**95% confidence interval**	***P***
**Total subjects**	33			
**CD56+ NKG2D+ (%)**				
< 59.0	16(48.5)	**0.127**	**0.024-0.675**	**0.015**
**BMI (Kg/m^2)**				
< 20.0	17(51.5)	0.187	0.027-1.299	0.090
**Age (years)**				
≥ 35	8(24.2)	0.162	0.012-2.213	0.173
**Age of menarche (years)**				
< 14	13(39.4)	0.558	0.081-3.828	0.553
**Maximum diameter of OE (cm)**				
≥ 7.0	12(36.4)	0.453	0.115-1.787	0.258
**Surgical approach**				
Laparoscope or hysteroscope*	23(69.7)	0.668	0.173-2.576	0.558
**Position of OE**				
Bilateral	6(18.2)	0.219	0.022-2.141	0.192
**Anti-Mullerian hormone (ng/ml)**				
< 4.65	15(45.5)	0.335	0.085-1.324	0.119

* Compared to those treated by laparoscope with hysteroscope.

Suppl. Table 4. Analysis of the percentage of CD56+ NKG2D+ cells and fertility in 68 controls during the study period.

**CD56+NKG2D+ (%)**	**Pregnancy/Cases (n)**	**MPT (days)**	**Log-Rank *P* Value**	**Cox regression**					
				**Model I[a]**		**Model II[b]**		**Model III[c]**	
				HR (95% *CI*)	*P*	HR (95% *CI*)	*P*	HR (95% *CI*)	*P*
≥ 59.0	25/49	126		1.00 (ref.)		1.00 (ref.)		1.00 (ref.)	
< 59.0	10/19	92	0.845	0.929(0.446-1.936)	0.845	0.917(0.440-1.911)	0.817	0.896(0.411-1.956)	0.783

Notes: MPT, median pregnancy time; HR, hazard ratio; CI, confidence interval; [a] no adjustment; [b] adjusting age; [c] adjusting the confounding factors including age, body mass index, and age at menarche.

Suppl. Table 5. Definition, markers and gating strategy of lymphocyte subgroups included in this study.

**Lymphocyte subgroups**	**Markers**	**Gating strategy**
T lymphocytes	CD3+	①+②
Th cells	CD3+CD4+	①+②+③
Tc cells	CD3+CD8+	①+②+④
Naïve CD4+T cells	CD3+CD4+CD45RA+CCR7+	①+②+③+(CD45RA+/CCR7+)
Terminally differentiated effector memory CD4+T cells	CD3+CD4+CD45RA+CCR7-	①+②+③+(CD45RA+/CCR7-)
Central memory CD4+T cells	CD3+CD4+CD45RA-CCR7+	①+②+③+(CD45RA-/CCR7+)
Effector memory CD4+T cells	CD3+CD4+CD45RA-CCR7-	①+②+③+(CD45RA-/CCR7-)
CD4(+)CD28(-) T cells	CD3+CD4+CD28-	①+②+③+(CD28-)
Naïve CD8+T cells	CD3+CD8+CCR7+CD45RA+	①+②+④+(CD45RA+/CCR7+)
Terminally differentiated effector memory CD8+T cells	CD3+CD8+CCR7-CD45RA+	①+②+④+(CD45RA+/CCR7-)
Central memory CD8+T cells	CD3+CD8+CCR7+CD45RA-	①+②+④+(CD45RA-/CCR7+)
Effector memory CD8+T cells	CD3+CD8+CCR7-CD45RA-	①+②+④+(CD45RA-/CCR7-)
CD8(+)CD28(-) T cells	CD3+CD8+CD28-	①+②+④+(CD28-)
Th1 cells	CD3+CD4+CXCR5-CXCR3+CCR4-	①+②+③+⑤+(CXCR3+/CCR4-)
Th2 cells	CD3+CD4+CXCR5-CXCR3-CCR4+	①+②+③+⑤+(CXCR3-/CCR4+)
Tc1 cells	CD3+CD8+CXCR5-CXCR3+CCR4-	①+②+④+⑤+(CXCR3+/CCR4-)
Tc2 cells	CD3+CD8+CXCR5-CXCR3-CCR4+	①+②+④+⑤+(CXCR3-/CCR4+)
Tfh cells	CD3+CD4+CXCR5+	①+②+③+⑥
Tfh1 cells	CD3+CD4+CXCR5+CXCR3+CCR4-	①+②+③+⑥+(CXCR3+/CCR4-)
Tfh2 cells	CD3+CD4+CXCR5+CXCR3-CCR4+	①+②+③+⑥+(CXCR3-/CCR4+)
NK cells	CD3-CD56+	①+⑦+⑧
CD56+KIR+ NK cells	CD3-CD56+CD94-KIR+	①+⑦+⑧+(CD94-/KIR+)
Activated NK cells	CD3-CD56+NKG2D+	①+⑦+⑧+(NKG2D+)
CD56+NKP30+ NK cells	CD3-CD56+NKP30+	①+⑦+⑧+(NKP30+)
CD56+NKP46+ NK cells	CD3-CD56+NKP46+	①+⑦+⑧+(NKP46+)
γδ T cells	CD3+gammadelta+	①+②+⑨
Vδ1+ γδ T cells	CD3+gammadelta+ Vdelta1+	①+②+⑨+(Vdelta1+)
Vδ2+ γδ T cells	CD3+gammadelta+ Vdelta2+	①+②+⑨+(Vdelta2+)

Notes: ①, Lymphocyte. ②,CD3+. ③, CD4+. ④, CD8+. ⑤, CXCR5-. ⑥, CXCR5+. ⑦, CD3-. ⑧,

CD56+. ⑨,   gammadelta+. "-/-", "+/-", "-/+" and "+/+" were defined as the quadrant of lower left, higher left, lower right and higher right respectively.

Suppl. Table 6. Comparision of blood tests between 33 OE patients and 68 controls without imputed data.

	**Mann-Whitney U**	***Z***	***P***
Hemoglobin	546.0	-3.273	0.001
Leukocytes	886.0	-0.424	0.671
Lymphocytes	565.5	-3.043	0.002
Erythrocytes	886.5	-0.420	0.674

Note: all listed items missing three patients.
